# Bis(2-amino­benzothia­zole-κ*N* ^1^)bis­(thio­cyanato-κ*N*)zinc(II)

**DOI:** 10.1107/S1600536809030931

**Published:** 2009-08-08

**Authors:** Seung Wook Suh, Chong-Hyeak Kim, Inn Hoe Kim

**Affiliations:** a Department of Chemistry, Konyang University, Nonsan 320-711, Republic of Korea; b Center for Chemical Analysis, Korea Research Institute of Chemical Technology, PO Box 107, Yuseong, Daejeon 305-600, Republic of Korea

## Abstract

The Zn^II^ ion in the title complex, [Zn(NCS)_2_(C_7_H_6_N_2_S)_2_], is tetra­hedrally coordinated within an N_4_ donor set defined by two N atoms of two terminal isothio­cyanate ligands and by two heterocyclic N atoms of two different 2-amino­benzothia­zole ligands. This arrangement is stabilized by intra­molecular N—H⋯N hydrogen bonds. In the crystal structure, mol­ecules are linked through N—H⋯S hydrogen bonds to form a two-dimensional array.

## Related literature

For related literature on organic–inorganic hybrid supra­molecular complexes, see: Batten & Robson (1998 ▶); Braga *et al.* (1998 ▶); Iwamoto (1996 ▶). For the use of pseudo-halides in the construction of supra­molecular assemblies, see: Vrieze & Koten (1987 ▶); Cortes *et al.* (1997 ▶); Yun *et al.* (2004 ▶); Kim *et al.* (2001 ▶, 2008 ▶). For the coordination chemistry of imidazole and thia­zole derivatives, see: Balch *et al.* (1993 ▶); Costes *et al.* (1991 ▶); Suh *et al.* (2005 ▶, 2007 ▶).
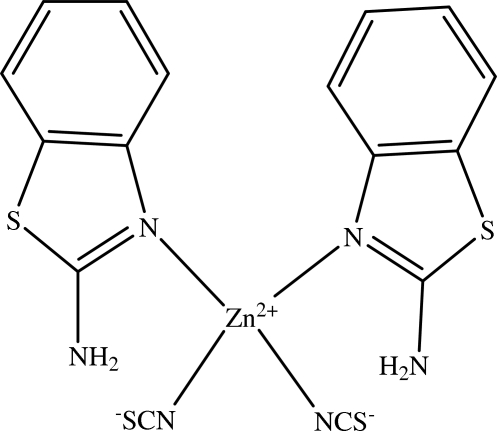


## Experimental

### 

#### Crystal data

[Zn(NCS)_2_(C_7_H_6_N_2_S)_2_]
*M* *_r_* = 481.93
Triclinic, 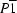

*a* = 8.4379 (1) Å
*b* = 9.4900 (1) Å
*c* = 13.3037 (2) Å
α = 97.735 (1)°
β = 107.302 (1)°
γ = 94.232 (1)°
*V* = 1000.52 (2) Å^3^
*Z* = 2
Mo *K*α radiation
μ = 1.66 mm^−1^
*T* = 296 K
0.41 × 0.28 × 0.21 mm

#### Data collection

Bruker SMART APEXII CCD area-detector diffractometer
Absorption correction: multi scan (*SADABS*; Bruker, 2001 ▶) *T*_min_ = 0.550, *T*_max_ = 0.722
19351 measured reflections
4901 independent reflections
4238 reflections with *I* > 2σ(*I*)
*R*_int_ = 0.028

#### Refinement

*R*[*F* ^2^ > 2σ(*F* ^2^)] = 0.030
*wR*(*F* ^2^) = 0.079
*S* = 1.05
4901 reflections
244 parameters
H-atom parameters constrained
Δρ_max_ = 0.63 e Å^−3^
Δρ_min_ = −0.60 e Å^−3^

### 

Data collection: *SMART* (Bruker, 2001 ▶); cell refinement: *SAINT* (Bruker, 2001 ▶); data reduction: *SAINT*; program(s) used to solve structure: *SHELXS97* (Sheldrick, 2008 ▶); program(s) used to refine structure: *SHELXL97* (Sheldrick, 2008 ▶); molecular graphics: *SHELXTL* (Sheldrick, 2008 ▶); software used to prepare material for publication: *SHELXTL*.

## Supplementary Material

Crystal structure: contains datablocks global, I. DOI: 10.1107/S1600536809030931/tk2521sup1.cif

Structure factors: contains datablocks I. DOI: 10.1107/S1600536809030931/tk2521Isup2.hkl

Additional supplementary materials:  crystallographic information; 3D view; checkCIF report

## Figures and Tables

**Table 1:** Hydrogen-bond geometry (Å, °)

*D*—H⋯*A*	*D*—H	H⋯*A*	*D*⋯*A*	*D*—H⋯*A*
N20—H20*A*⋯N1	0.86	2.24	3.027 (3)	152
N20—H20*B*⋯S1^i^	0.86	2.70	3.5015 (19)	156
N30—H30*A*⋯N2	0.86	2.21	3.002 (3)	152
N30—H30*B*⋯S2^ii^	0.86	2.57	3.404 (2)	162

Symmetry codes: (i) 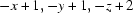; (ii) 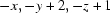.

**Fig. 1.**
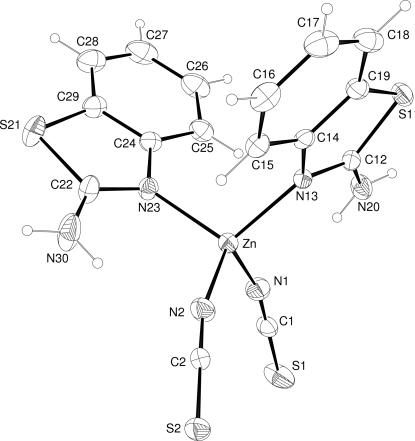
The molecular structure of (I), showing 30% probability displacement ellipsoids and the atom-numbering scheme.

## supplementary crystallographic information

### Comment

Organic-inorganic hybrid supramolecular complexes of 1-, 2-, and 3-D frameworks has attracted great interest recently (Iwamoto, 1996; Batten & Robson, 1998), as they have useful properties, viz. electronic, magnetic, optical, catalytic, *etc.* (Braga *et al.*, 1998). For designing novel multi-dimensional frameworks, we (Kim *et al.*, 2001; Kim *et al.*, 2008) and others (Cortes *et al.*, 1997; Yun *et al.*, 2004) have used the coordination properties of various pseudohalide ions and complementary organic ligands. Pseudo-halide ions, *e.g.* CN^-^, SCN^-^, N_3_^-^, are known to build up 1-, 2- and 3-D structures by bridging metal centers (Vrieze & Koten, 1987). The of use of complementary organic ligands, such as aliphatic and aromatic amines is also known to play an important role in stabilizing multi-dimensional structures. In particulae, aromatic heterocycles such as imidazole and thiazole derivatives represent an important class of ligands in coordination chemistry (Balch *et al.*, 1993; Costes *et al.*, 1991). However, frameworks of metal complexes containing thiazole derivatives have been considerably less investigated. Our research is focused on the development of novel supramolecular framework structures utilizing the terminal and bridging properties of pseudo-halide ions, and the coordination behaviour of thiazole derivatives as complementary organic ligands (Suh *et al.*, 2005, 2007). Herein, we present the synthesis and structure determination of the title complex, (I), with 2-aminobenzothiazole, Fig. 1.

### Experimental

A water-methanolic (1:1) solution (20 ml) of potassium thiocyanate (2 mmol, 0.19 g) was added to a water-methanolic (1:1) solution (20 ml) of Zn(NO_3_)_2_.6H_2_O (1 mmol, 0.30 g). To this mixture, a water-methanolic (1:1) solution (20 ml) of 2-aminobenzothiazole (3 mmol, 0.45 g) was introduced, with stirring. The small amount of precipitates formed from the resulting solution were filtered off. The filtered solution was allowed to stand at room temperature. After a few days silver blocks were obtained. Elemental analysis found: C 40.41, H 2.67, N 18.11, S 26.59, Zn 13.60%; C_16_H_12_N_6_S_4_Zn requires: C 39.87, H 2.51, N 17.44, S 26.61, Zn 13.56%.

### Refinement

Positional parameters for the H atoms were calculated geometrically and constrained to ride on their attached atoms with C—H = 0.93 Å and N—H = 0.86 Å, and with *U*_iso_(H) = 1.2*U*_eq_(C, N).

### Crystal data

**Table:**

[Zn(NCS)_2_(C_7_H_6_N_2_S)_2_]	*Z* = 2
*M**_r_* = 481.93	*F*(000) = 488
Triclinic, *P*1	*D*_x_ = 1.600 Mg m^−^^3^*D*_m_ = 1.59 Mg m^−^^3^*D*_m_ measured by flotation method
Hall symbol: -P 1	Mo *K*α radiation, λ = 0.71073 Å
*a* = 8.4379 (1) Å	Cell parameters from 9879 reflections
*b* = 9.4900 (1) Å	θ = 2.5–28.1°
*c* = 13.3037 (2) Å	µ = 1.66 mm^−^^1^
α = 97.735 (1)°	*T* = 296 K
β = 107.302 (1)°	Block, silver
γ = 94.232 (1)°	0.41 × 0.28 × 0.21 mm
*V* = 1000.52 (2) Å^3^	

### Data collection

**Table:**

Bruker SMART APEXII CCD area-detector diffractometer	4901 independent reflections
Radiation source: fine-focus sealed tube	4238 reflections with *I* > 2σ(*I*)
graphite	*R*_int_ = 0.028
φ and ω scans	θ_max_ = 28.3°, θ_min_ = 1.6°
Absorption correction: multi scan (*SADABS*; Bruker, 2001)	*h* = −11→11
*T*_min_ = 0.550, *T*_max_ = 0.722	*k* = −12→12
19351 measured reflections	*l* = −17→17

### Refinement

**Table:**

Refinement on *F*^2^	Primary atom site location: structure-invariant direct methods
Least-squares matrix: full	Secondary atom site location: difference Fourier map
*R*[*F*^2^ > 2σ(*F*^2^)] = 0.030	Hydrogen site location: inferred from neighbouring sites
*wR*(*F*^2^) = 0.079	H-atom parameters constrained
*S* = 1.05	*w* = 1/[σ^2^(*F*_o_^2^) + (0.0335*P*)^2^ + 0.369*P*] where *P* = (*F*_o_^2^ + 2*F*_c_^2^)/3
4901 reflections	(Δ/σ)_max_ = 0.001
244 parameters	Δρ_max_ = 0.63 e Å^−^^3^
0 restraints	Δρ_min_ = −0.60 e Å^−^^3^

### Special details

**Table:**

Geometry. All e.s.d.'s (except the e.s.d. in the dihedral angle between two l.s. planes) are estimated using the full covariance matrix. The cell e.s.d.'s are taken into account individually in the estimation of e.s.d.'s in distances, angles and torsion angles; correlations between e.s.d.'s in cell parameters are only used when they are defined by crystal symmetry. An approximate (isotropic) treatment of cell e.s.d.'s is used for estimating e.s.d.'s involving l.s. planes.
Refinement. Refinement of *F*^2^ against ALL reflections. The weighted *R*-factor *wR* and goodness of fit *S* are based on *F*^2^, conventional *R*-factors *R* are based on *F*, with *F* set to zero for negative *F*^2^. The threshold expression of *F*^2^ > σ(*F*^2^) is used only for calculating *R*-factors(gt) *etc*. and is not relevant to the choice of reflections for refinement. *R*-factors based on *F*^2^ are statistically about twice as large as those based on *F*, and *R*- factors based on ALL data will be even larger.

### Fractional atomic coordinates and isotropic or equivalent isotropic displacement parameters (Å^2^)

**Table:**

	*x*	*y*	*z*	*U*_iso_*/*U*_eq_	
Zn	0.33334 (3)	0.77964 (2)	0.744958 (17)	0.04196 (8)	
S1	0.11604 (9)	0.33772 (8)	0.79681 (7)	0.0884 (3)	
C1	0.2117 (3)	0.4851 (2)	0.78611 (17)	0.0523 (5)	
N1	0.2812 (2)	0.5896 (2)	0.77780 (16)	0.0613 (5)	
S2	−0.15336 (6)	0.99430 (6)	0.72597 (4)	0.05379 (13)	
C2	0.0121 (2)	0.9254 (2)	0.72033 (14)	0.0431 (4)	
N2	0.1339 (2)	0.8781 (2)	0.71756 (15)	0.0588 (5)	
S11	0.80227 (6)	0.93870 (7)	1.02664 (4)	0.05593 (14)	
C12	0.6289 (2)	0.8274 (2)	0.94188 (15)	0.0439 (4)	
N13	0.52630 (18)	0.88677 (16)	0.86862 (12)	0.0395 (3)	
C14	0.5820 (2)	1.0329 (2)	0.87934 (15)	0.0412 (4)	
C15	0.5032 (3)	1.1285 (2)	0.81714 (17)	0.0511 (5)	
H15A	0.4031	1.0990	0.7625	0.061*	
C16	0.5770 (3)	1.2695 (2)	0.8383 (2)	0.0650 (6)	
H16A	0.5260	1.3349	0.7968	0.078*	
C17	0.7250 (4)	1.3142 (3)	0.9199 (2)	0.0728 (7)	
H17A	0.7721	1.4091	0.9322	0.087*	
C18	0.8037 (3)	1.2210 (3)	0.9829 (2)	0.0666 (6)	
H18A	0.9029	1.2515	1.0381	0.080*	
C19	0.7310 (2)	1.0799 (2)	0.96185 (16)	0.0495 (5)	
N20	0.6088 (2)	0.6899 (2)	0.95306 (15)	0.0591 (5)	
H20A	0.5240	0.6334	0.9106	0.071*	
H20B	0.6807	0.6578	1.0027	0.071*	
S21	0.44673 (11)	0.79917 (8)	0.43370 (5)	0.0764 (2)	
C22	0.3431 (3)	0.8192 (3)	0.52843 (18)	0.0590 (5)	
N23	0.4067 (2)	0.76110 (17)	0.61379 (13)	0.0458 (4)	
C24	0.5455 (2)	0.6922 (2)	0.60632 (16)	0.0468 (4)	
C25	0.6394 (3)	0.6195 (2)	0.6816 (2)	0.0583 (5)	
H25A	0.6128	0.6118	0.7438	0.070*	
C26	0.7738 (3)	0.5585 (3)	0.6631 (3)	0.0790 (8)	
H26A	0.8379	0.5088	0.7131	0.095*	
C27	0.8139 (4)	0.5707 (4)	0.5707 (3)	0.0895 (10)	
H27A	0.9055	0.5296	0.5599	0.107*	
C28	0.7221 (4)	0.6415 (3)	0.4953 (3)	0.0801 (8)	
H28A	0.7495	0.6489	0.4333	0.096*	
C29	0.5858 (3)	0.7027 (2)	0.51366 (18)	0.0580 (5)	
N30	0.2092 (3)	0.8888 (3)	0.51170 (19)	0.0971 (9)	
H30A	0.1575	0.8984	0.5586	0.116*	
H30B	0.1741	0.9243	0.4540	0.116*	

### Atomic displacement parameters (Å^2^)

**Table:**

	*U*^11^	*U*^22^	*U*^33^	*U*^12^	*U*^13^	*U*^23^
Zn	0.03854 (12)	0.04733 (13)	0.03935 (13)	0.00595 (9)	0.01096 (9)	0.00711 (9)
S1	0.0599 (4)	0.0743 (4)	0.1198 (6)	−0.0084 (3)	−0.0023 (4)	0.0550 (4)
C1	0.0425 (10)	0.0574 (11)	0.0531 (12)	0.0052 (9)	0.0048 (8)	0.0190 (9)
N1	0.0610 (11)	0.0563 (10)	0.0661 (12)	−0.0027 (9)	0.0190 (9)	0.0155 (9)
S2	0.0406 (2)	0.0668 (3)	0.0544 (3)	0.0127 (2)	0.0152 (2)	0.0070 (2)
C2	0.0407 (9)	0.0515 (10)	0.0338 (9)	0.0031 (8)	0.0079 (7)	0.0053 (7)
N2	0.0448 (9)	0.0739 (12)	0.0547 (11)	0.0161 (8)	0.0120 (8)	0.0036 (9)
S11	0.0412 (3)	0.0730 (3)	0.0447 (3)	0.0075 (2)	0.0039 (2)	0.0007 (2)
C12	0.0407 (9)	0.0573 (11)	0.0344 (9)	0.0084 (8)	0.0123 (7)	0.0076 (8)
N13	0.0372 (7)	0.0487 (8)	0.0335 (8)	0.0062 (6)	0.0115 (6)	0.0077 (6)
C14	0.0392 (9)	0.0484 (9)	0.0403 (10)	0.0048 (7)	0.0211 (7)	0.0025 (7)
C15	0.0553 (12)	0.0523 (11)	0.0507 (12)	0.0096 (9)	0.0230 (9)	0.0094 (9)
C16	0.0811 (17)	0.0519 (12)	0.0756 (16)	0.0145 (11)	0.0418 (14)	0.0134 (11)
C17	0.0772 (17)	0.0495 (12)	0.097 (2)	−0.0037 (12)	0.0448 (15)	−0.0048 (13)
C18	0.0529 (12)	0.0634 (14)	0.0774 (17)	−0.0043 (11)	0.0255 (12)	−0.0152 (12)
C19	0.0417 (10)	0.0582 (11)	0.0483 (11)	0.0033 (8)	0.0195 (8)	−0.0032 (9)
N20	0.0611 (11)	0.0609 (10)	0.0503 (11)	0.0088 (9)	0.0041 (8)	0.0213 (8)
S21	0.1069 (6)	0.0854 (4)	0.0525 (4)	0.0214 (4)	0.0440 (4)	0.0158 (3)
C22	0.0754 (15)	0.0659 (13)	0.0435 (12)	0.0224 (11)	0.0250 (10)	0.0133 (10)
N23	0.0508 (9)	0.0488 (8)	0.0403 (9)	0.0130 (7)	0.0164 (7)	0.0072 (7)
C24	0.0438 (10)	0.0428 (9)	0.0506 (11)	0.0013 (8)	0.0161 (8)	−0.0041 (8)
C25	0.0482 (11)	0.0591 (12)	0.0647 (14)	0.0136 (9)	0.0143 (10)	0.0034 (10)
C26	0.0526 (13)	0.0797 (17)	0.097 (2)	0.0223 (12)	0.0147 (13)	−0.0010 (15)
C27	0.0536 (15)	0.097 (2)	0.112 (3)	0.0153 (14)	0.0316 (16)	−0.0227 (19)
C28	0.0709 (17)	0.0879 (18)	0.0829 (19)	−0.0006 (14)	0.0436 (15)	−0.0206 (15)
C29	0.0603 (13)	0.0569 (12)	0.0562 (13)	−0.0002 (10)	0.0271 (10)	−0.0092 (10)
N30	0.116 (2)	0.142 (2)	0.0636 (15)	0.0820 (19)	0.0421 (14)	0.0543 (15)

### Geometric parameters (Å, °)

**Table:**

Zn—N2	1.9482 (18)	C18—C19	1.387 (3)
Zn—N1	1.9610 (18)	C18—H18A	0.9300
Zn—N23	2.0089 (16)	N20—H20A	0.8600
Zn—N13	2.0257 (15)	N20—H20B	0.8600
S1—C1	1.607 (2)	S21—C22	1.733 (2)
C1—N1	1.150 (3)	S21—C29	1.739 (3)
S2—C2	1.602 (2)	C22—N23	1.315 (3)
C2—N2	1.160 (3)	C22—N30	1.328 (3)
S11—C12	1.731 (2)	N23—C24	1.405 (2)
S11—C19	1.738 (2)	C24—C25	1.379 (3)
C12—N13	1.317 (2)	C24—C29	1.387 (3)
C12—N20	1.337 (3)	C25—C26	1.381 (3)
N13—C14	1.406 (2)	C25—H25A	0.9300
C14—C15	1.383 (3)	C26—C27	1.386 (5)
C14—C19	1.396 (3)	C26—H26A	0.9300
C15—C16	1.389 (3)	C27—C28	1.361 (5)
C15—H15A	0.9300	C27—H27A	0.9300
C16—C17	1.382 (4)	C28—C29	1.396 (3)
C16—H16A	0.9300	C28—H28A	0.9300
C17—C18	1.372 (4)	N30—H30A	0.8600
C17—H17A	0.9300	N30—H30B	0.8600
			
N2—Zn—N1	109.42 (9)	C18—C19—S11	128.30 (19)
N2—Zn—N23	108.31 (8)	C14—C19—S11	110.17 (15)
N1—Zn—N23	110.24 (8)	C12—N20—H20A	120.0
N2—Zn—N13	112.85 (7)	C12—N20—H20B	120.0
N1—Zn—N13	108.15 (7)	H20A—N20—H20B	120.0
N23—Zn—N13	107.85 (6)	C22—S21—C29	89.42 (11)
N1—C1—S1	179.1 (2)	N23—C22—N30	124.7 (2)
C1—N1—Zn	163.33 (19)	N23—C22—S21	115.26 (17)
N2—C2—S2	178.5 (2)	N30—C22—S21	119.99 (18)
C2—N2—Zn	165.33 (19)	C22—N23—C24	111.00 (17)
C12—S11—C19	89.28 (10)	C22—N23—Zn	126.00 (15)
N13—C12—N20	124.72 (18)	C24—N23—Zn	122.79 (13)
N13—C12—S11	115.89 (15)	C25—C24—C29	120.2 (2)
N20—C12—S11	119.39 (15)	C25—C24—N23	125.73 (19)
C12—N13—C14	110.52 (16)	C29—C24—N23	114.10 (19)
C12—N13—Zn	125.21 (13)	C24—C25—C26	118.8 (2)
C14—N13—Zn	123.77 (12)	C24—C25—H25A	120.6
C15—C14—C19	119.82 (18)	C26—C25—H25A	120.6
C15—C14—N13	126.06 (18)	C25—C26—C27	120.5 (3)
C19—C14—N13	114.12 (17)	C25—C26—H26A	119.7
C14—C15—C16	118.4 (2)	C27—C26—H26A	119.7
C14—C15—H15A	120.8	C28—C27—C26	121.4 (3)
C16—C15—H15A	120.8	C28—C27—H27A	119.3
C17—C16—C15	121.1 (2)	C26—C27—H27A	119.3
C17—C16—H16A	119.5	C27—C28—C29	118.2 (3)
C15—C16—H16A	119.5	C27—C28—H28A	120.9
C18—C17—C16	121.2 (2)	C29—C28—H28A	120.9
C18—C17—H17A	119.4	C24—C29—C28	120.9 (3)
C16—C17—H17A	119.4	C24—C29—S21	110.20 (16)
C17—C18—C19	118.0 (2)	C28—C29—S21	128.9 (2)
C17—C18—H18A	121.0	C22—N30—H30A	120.0
C19—C18—H18A	121.0	C22—N30—H30B	120.0
C18—C19—C14	121.5 (2)	H30A—N30—H30B	120.0
			
N2—Zn—N1—C1	16.6 (7)	N13—C14—C19—S11	0.15 (19)
N23—Zn—N1—C1	−102.4 (7)	C12—S11—C19—C18	179.7 (2)
N13—Zn—N1—C1	139.9 (7)	C12—S11—C19—C14	−0.90 (14)
N1—Zn—N2—C2	44.1 (7)	C29—S21—C22—N23	0.9 (2)
N23—Zn—N2—C2	164.3 (7)	C29—S21—C22—N30	−178.9 (2)
N13—Zn—N2—C2	−76.3 (7)	N30—C22—N23—C24	178.5 (2)
C19—S11—C12—N13	1.57 (15)	S21—C22—N23—C24	−1.3 (3)
C19—S11—C12—N20	−179.42 (17)	N30—C22—N23—Zn	−6.6 (4)
N20—C12—N13—C14	179.31 (18)	S21—C22—N23—Zn	173.67 (10)
S11—C12—N13—C14	−1.7 (2)	N2—Zn—N23—C22	7.0 (2)
N20—C12—N13—Zn	−8.6 (3)	N1—Zn—N23—C22	126.68 (19)
S11—C12—N13—Zn	170.34 (8)	N13—Zn—N23—C22	−115.45 (19)
N2—Zn—N13—C12	139.20 (15)	N2—Zn—N23—C24	−178.63 (14)
N1—Zn—N13—C12	18.00 (17)	N1—Zn—N23—C24	−58.93 (16)
N23—Zn—N13—C12	−101.21 (15)	N13—Zn—N23—C24	58.94 (15)
N2—Zn—N13—C14	−49.73 (15)	C22—N23—C24—C25	−179.3 (2)
N1—Zn—N13—C14	−170.92 (13)	Zn—N23—C24—C25	5.6 (3)
N23—Zn—N13—C14	69.87 (14)	C22—N23—C24—C29	1.1 (3)
C12—N13—C14—C15	−178.80 (18)	Zn—N23—C24—C29	−174.00 (14)
Zn—N13—C14—C15	9.0 (2)	C29—C24—C25—C26	0.3 (3)
C12—N13—C14—C19	1.0 (2)	N23—C24—C25—C26	−179.3 (2)
Zn—N13—C14—C19	−171.23 (12)	C24—C25—C26—C27	0.3 (4)
C19—C14—C15—C16	0.8 (3)	C25—C26—C27—C28	−0.6 (5)
N13—C14—C15—C16	−179.38 (18)	C26—C27—C28—C29	0.3 (4)
C14—C15—C16—C17	−0.5 (3)	C25—C24—C29—C28	−0.6 (3)
C15—C16—C17—C18	−0.2 (4)	N23—C24—C29—C28	179.0 (2)
C16—C17—C18—C19	0.5 (4)	C25—C24—C29—S21	179.88 (16)
C17—C18—C19—C14	−0.1 (3)	N23—C24—C29—S21	−0.5 (2)
C17—C18—C19—S11	179.27 (18)	C27—C28—C29—C24	0.3 (4)
C15—C14—C19—C18	−0.6 (3)	C27—C28—C29—S21	179.8 (2)
N13—C14—C19—C18	179.61 (18)	C22—S21—C29—C24	−0.17 (17)
C15—C14—C19—S11	179.95 (14)	C22—S21—C29—C28	−179.7 (2)

### Hydrogen-bond geometry (Å, °)

**Table:**

*D*—H···*A*	*D*—H	H···*A*	*D*···*A*	*D*—H···*A*
N20—H20A···N1	0.86	2.24	3.027 (3)	152
N20—H20B···S1^i^	0.86	2.70	3.5015 (19)	156
N30—H30A···N2	0.86	2.21	3.002 (3)	152
N30—H30B···S2^ii^	0.86	2.57	3.404 (2)	162

Symmetry codes: (i) −*x*+1, −*y*+1, −*z*+2; (ii) −*x*, −*y*+2, −*z*+1.
